# FBXW7 attenuates tumor drug resistance and enhances the efficacy of immunotherapy

**DOI:** 10.3389/fonc.2023.1147239

**Published:** 2023-03-14

**Authors:** Shimin Chen, Jichun Lin, Jiaojiao Zhao, Qian Lin, Jia Liu, Qiang Wang, Ryan Mui, Leina Ma

**Affiliations:** ^1^ Department of Oncology, Cancer Institute, The Affiliated Hospital of Qingdao University, Qingdao, China; ^2^ Qingdao Cancer Institute, Qingdao, China; ^3^ School of Basic Medicine, Qingdao University, Qingdao, China; ^4^ Department of Pharmacology, School of Pharmacy, Qingdao University, Qingdao, China; ^5^ Oncology Department, Shandong Second Provincial General Hospital, Jinan, China; ^6^ Department of Gastroenterology, Sparrow Hospital, Lansing, MI, United States

**Keywords:** FBXW7, drug resistance, E3 ligase, cancer, immunotherapy

## Abstract

FBXW7 (F-box and WD repeat domain containing 7) is a critical subunit of the Skp1-Cullin1-F-box protein (SCF), acting as an E3 ubiquitin ligase by ubiquitinating targeted protein. Through degradation of its substrates, FBXW7 plays a pivotal role in drug resistance in tumor cells and shows the potential to rescue the sensitivity of cancer cells to drug treatment. This explains why patients with higher FBXW7 levels exhibit higher survival times and more favorable prognosis. Furthermore, FBXW7 has been demonstrated to enhance the efficacy of immunotherapy by targeting the degradation of specific proteins, as compared to the inactivated form of FBXW7. Additionally, other F-box proteins have also shown the ability to conquer drug resistance in certain cancers. Overall, this review aims to explore the function of FBXW7 and its specific effects on drug resistance in cancer cells.

## 1 Introduction

Based on their mechanisms, drug resistance can be categorized into two types: primary and acquired drug resistance (1).

Primary resistance occurs during the initial stages of treatment when the tumor shows no response to the therapy. This is due to either pre-existing genetic alterations or the rapid adaptation of tumor cells to therapy (2). Tumor cells with pre-existing genetic alteration are not affected by drugs, and therefore, their oncogenic signaling pathways continue to function normally. Tumor cells that rapidly adapt to therapy may present rewiring of the oncogenic signaling pathway once the drug takes effect and suppresses the signaling pathway, leading to drug resistance (2).

Acquired drug resistance can be induced by three mechanisms, including genetic drivers, activation of bypass signaling, and histologic transformation (2). In the first mechanism, the activity of driver oncogenes confers drug resistance on cancer cells. Driver oncogenes are triggered by gene mutations, gene amplification, and gene fusions, leading to the activation of downstream signaling pathways involving mitogen-activated protein kinase (MAPK) and phosphoinositide 3 kinase (PI3K) (3). In the second mechanism, “bypass” signaling occurs in tumor cells, suppressing the targeted pathway of drugs (2). The third mechanism is histologic transformation, such as squamous transformation, which is one of the primary reasons for resistance to First-line Osimertinib in EGFR-mutant lung cancer (4).

The E3 ligase plays a crucial role in the process of protein ubiquitination by transferring ubiquitin to the lysine residues of the substrates with the cooperation of the E1 and E2 enzymes. Additionally, the E3 ligase is involved in cellular non-degradable functions such as DNA repair, metabolism, and protein complex assembly (5). Dysfunction of the E3 ligase caused by its mutation regulates many signaling pathways, promoting the development of cancer (5).

**Figure 1:**
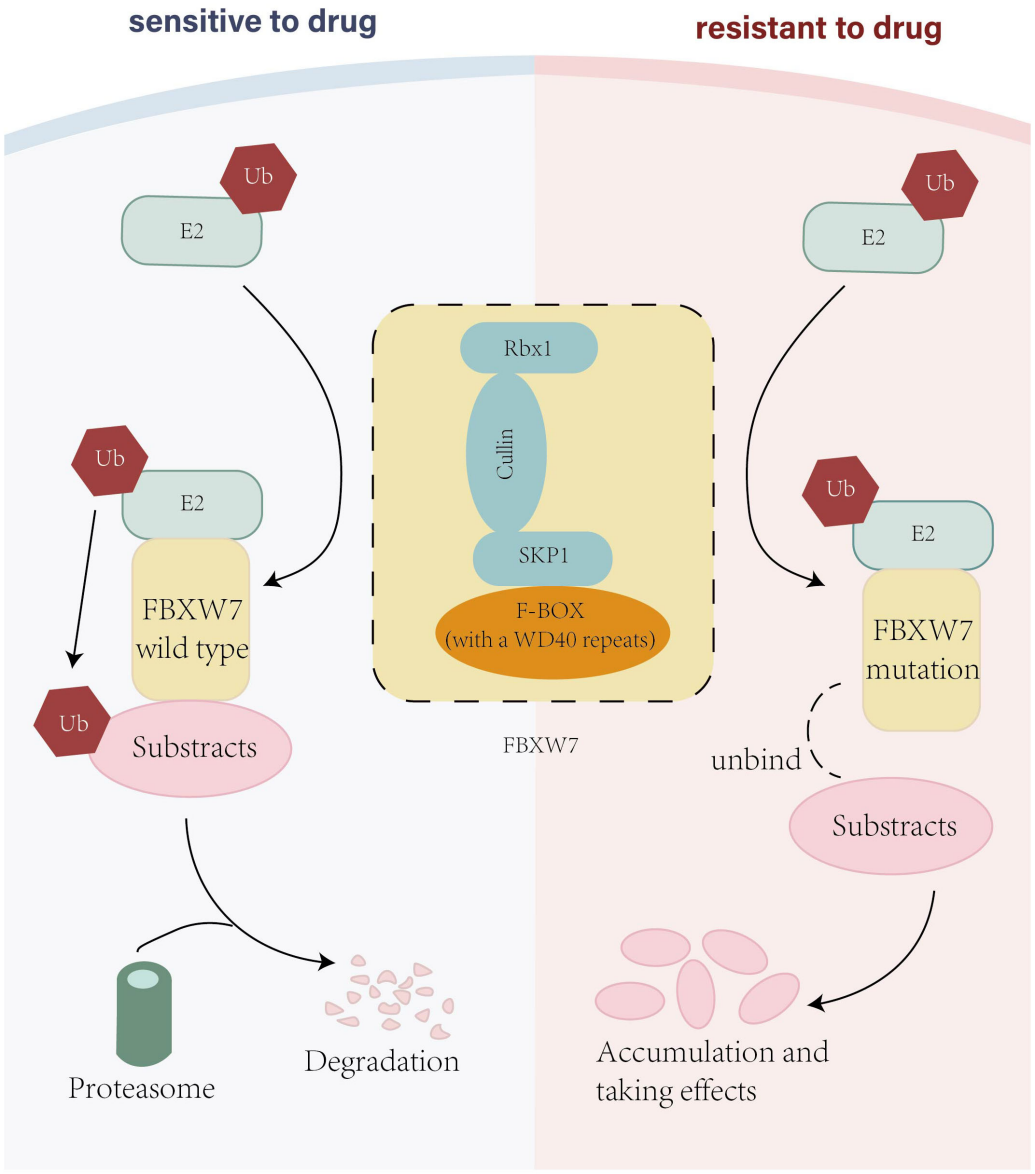
The figure shows the construction of FBXW7 and compares the way that FBXW7 wild type and FBXW7 mutation led to drug sensitivity and drug resistance respectively.

As an E3-ligase, the SCF complex ubiquitinates targeted proteins, establishing the foundation for subsequent degradation. The SCF complex comprises four constructions: Skp1, Cul-1, Rbx1, and an F-box protein (6). Based on the variable types of interaction domains binding to substrates, F-box could be classified into three isoforms: FBXW(interaction domains are WD40 repeats), FBXL(interaction domains are leucine-rich repeats), and FBXO(interaction domains are “others”) (7). The primary function of FBXW7 is to recognize and ubiquity targeted proteins, providing conditions for proteasome to identify the targeted protein and eventually degrade them (**Figure 1**).

In various cancers, FBXW7 is determined to have an effect. For example, the lack of FBXW7 in mice enhances cancer metastasis in both the cell-autonomous and the non-cell-autonomous ways (8). It is also found that FBXW7 suppresses gastric cancer (GC) metastasis by inducing Brg1 degradation (9). Both *in vitro* and *in vivo* studies reveal that the knockdown of FBXW7 enhances the invasion and migration in GC (10). In addition, FBXW7 is also associated with the prognosis of the patients, including the survival of post-operative patients who had colorectal liver metastases (11). Colorectal liver metastases patients with high FBXW7 have better disease-free survival. FBXW7 deficiency is associated with poor prognosis in human ovarian cancer as well (12). A clinical study has concluded that the 3 years of disease-free survival of the low and high Fbxw7 groups were 12.5% and 47.0%, respectively (13). What’s more, some experiments using FBXW7 knockout mouse models constructed by knocking out the gene in embryonic stem cells have also demonstrated that abnormal expression of FBXW7 leads to tumor formation (14).

**Figure 2:**
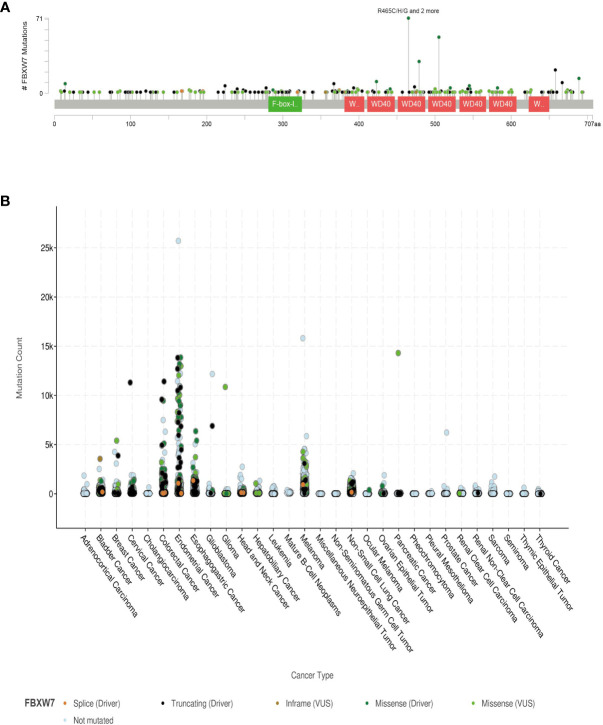
The situation of FBXW7 mutation is performed using cBioportal on the TCGA database (http://www.cbioportal.org/) **(A)** Mutation sites of FBXW7 and their mutation types. Different colors of spots indicate mutation types. Green spots represent missense mutation; black spots represent truncating mutation; brown spots represent splice mutation; purple spots represent struction variation or fusion mutation. **(B)** Mutation counts of FBXW7 in various cancer types. The color of these dots represent various type of mutation. This coordinate axis shows 30 types of cancers.

Among all subtypes of the FBXW family, the FBXW7 mutation occurs most frequently in cancers (6). In all types of FBXW7 mutations that occurred in malignant tumors, missense substitutions account for 72.70%, while nonsense substitutions and insertion/deletion mutations occur 13.82% and 7.89%, respectively (15). Endometrial carcinoma, colorectal adenocarcinoma, and esophagogastric adenocarcinoma are the top three tumors with the highest incidence of FBXW7 mutations (15). According to the data of the online database, FBXW7 can mutate at multiple sites, and FBXW7 mutations can be found in various tumors (**Figures 2A, B**).

## 2 FBXW7 ubiquitinates its downstream substrates to regulate drug resistance

**Figure 3:**
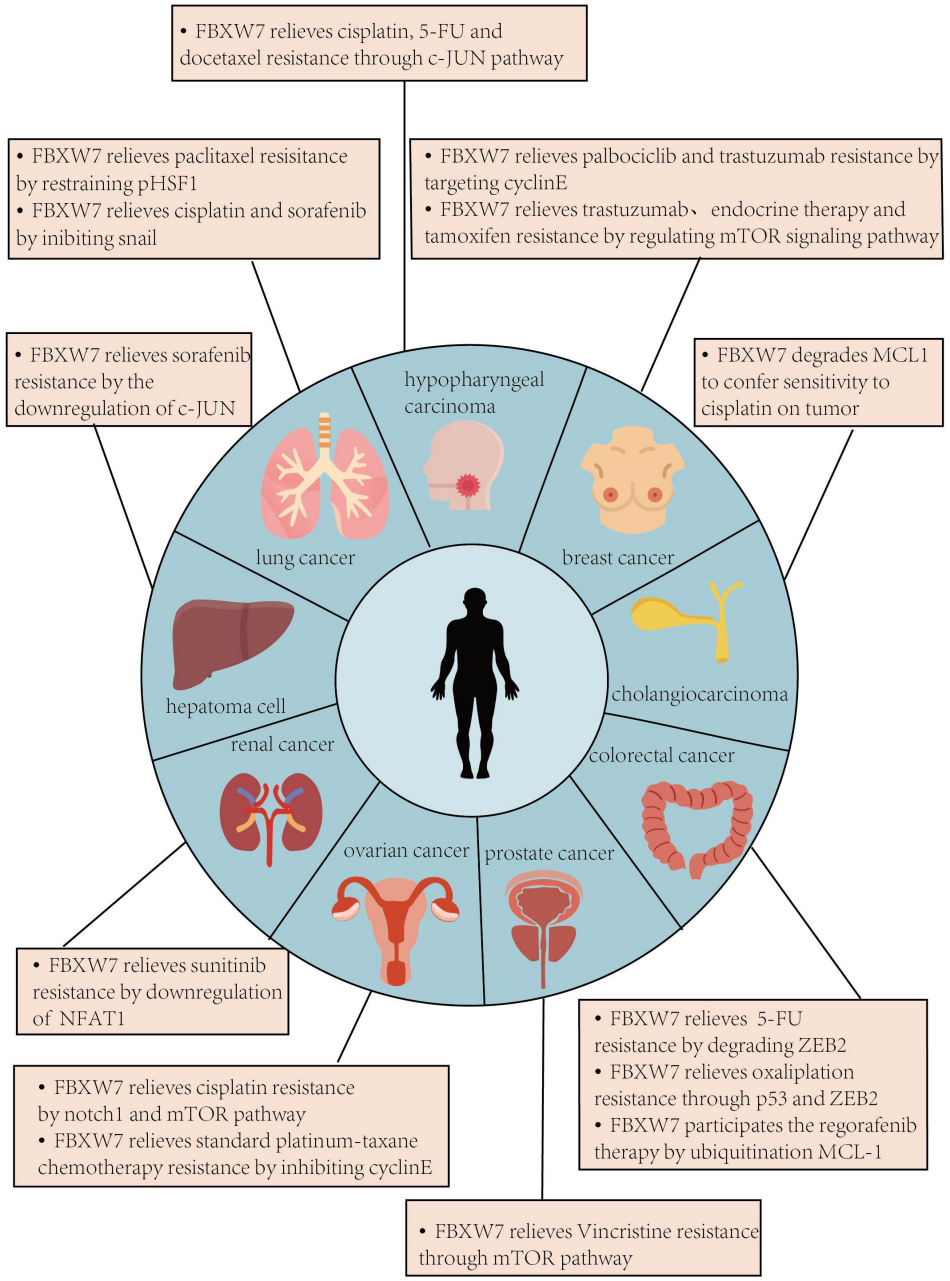
This figure indicates the role of FBXW7 on drug resistance in different tumors. In different tumors, the substrates of FBXW7 which take effects are not exactly the same, and the types of drugs whose sensitivity is regulated by FBXW7 also vary among different tumors.

FBXW7 has a range of target substrates, which are subject to regulation by the E3 ligase through ubiquitination. Most of these substrates are well-known oncogenic proteins frequently overexpressed in various human cancers, leading to FBXW7 being considered a tumor suppressor that negatively regulates these proteins (16). Through these substrates and corresponding pathways, FBXW7 has been found to have positive effects on drug resistance (**Supplementary Table 1**). Notably, the influence of FBXW7 is not limited to a specific tumor or drugs; rather, it can be observed in a broad spectrum of tumor treatments. The effects of FBXW7 on drug resistance are mediated by its ubiquitination of downstream substrates (**Figure 3**).

### 2.1 FBXW7 regulates drug resistance by degrading transcription factors

c-MYC, a member of the MYC family, plays a critical role in regulating cell proliferation, apoptosis, and metabolism. Studies have shown that medulloblastoma cells with a FBXW7 mutant (T205D) exhibit a higher level of c-MYC signal compared to those with wild-type FBXW7. Overexpression of FBXW7 enhances the apoptosis of the medulloblastoma cells. However, FBXW7 levels are significantly reduced in medulloblastoma, contributing to drug resistance (17). Likewise, decreased levels of FBXW7 in T-ALL cell lines promote tolerance to a γ-secretase inhibitor through the higher expression of c-MYC (18). c-MYC is also involved in regulation of drug resistance in various cancers, such as endometrial cancer, where it induces drug resistance by upregulating the expression of ATP-binding cassette subfamily B member 1 (ABCB1) (19).In glioma stem cells, knocking down c-MYC causes cell cycle arrest and promotes apoptosis, thereby decreasing glioma resistance to drugs (20).

c-Jun, a highly unstable transcription factor, has been shown to play a role in drug resistance in hypopharyngeal carcinoma and human hepatoma cells. *In vivo* experiments demonstrate that patients resistant to docetaxel/cisplatin/5-fluorouracil (TPF) have the upregulation of c-Jun in their hypopharyngeal carcinoma tissues (21), while overexpression of c-Jun inhibits human hepatoma cells from being sensitive to sorafenib during treatment (22). After being phosphorylated by glycogen synthase kinase-3 (GSK3), c-Jun can be recognized by FBXW7 and subsequently degraded. *In vitro* studies have proved that the deletion of FBXW7 leads to c-Jun accumulation (23). c-Jun may also indirectly cause drug resistance through other proteins, such as WEE1, which is a cell cycle regulator. c-Jun promotes WEE1 expression, protecting cisplatin-exposed ovarian cancer cells from cell cycle arrest and leading to the survival and proliferation of cisplatin-resistant cells (24).

Phosphorylated HSF1 participates in regulating multidrug resistance in human lung cancer cells, promoting paclitaxel sensitivity (25). Although the mRNA level of HSF does not show remarkable changes in drug resistance cancer cells, post-translational modification indicates a positive impact on Multidrug Sensitivity (MDR1) (25). The overexpressed FBXW7 combines with pHSF1 at Ser303/307, promoting its degradation. In uterine cancer sarcoma cells, HSF1 knockdown increases lipid ROS and iron levels, inhibits cell growth, and promotes sensitivity to doxorubicin and gemcitabine treatment. Targeting HSF1 thus reverses drug resistance of doxorubicin and gemcitabine in uterine cancer sarcoma *via* the ferroptosis pathway (26).

The high expression of Nuclear Factors of Activated T-cell (NFAT) is a common feature of many human cancers, and it has been linked to tumor growth, sunitinib resistance, and improved PD-L1 expression *via* the PI3K/AKT/GSK-3β signaling pathway (27). FBXW7 induces NFAT1 degradation, leading to PD-L1 downregulation and subsequent immunoreaction, which is correlated with prognosis (28). NFAT proteins play many roles in tumors, including participating in cancer cell proliferation, inhibiting cell apoptosis, inducing invasion and migration, etc. In addition, NFAT proteins can also induce drug resistance through calcineurin-dependent and independent pathways (29).

Snail1, a transcription factor involved in Epithelial-Mesenchymal Transition(EMT), combines and inhibits the E­cadherin promoter to suppress cell adhesion and enhance cell migration (30). During EMT, epithelial cells transfer into motile mesenchymal cells (31). This phenomenon plays a crucial role in tumor metastasis, stem cell phenotype of cancer, and chemo-resistance (32). Interestingly, in the NSCLC tissues, FBXW7 negatively impacts Snail1’s protein levels but not its mRNA levels (30). The restoration of Snail1 could confer drug resistance on the FBXW7-silenced human lung cancer cells, particularly to cisplatin and sorafenib, by promoting EMT (30).

ZEB2 is another transcription factor involved in EMT, promotes EMT and cell invasion in colorectal cancer (CRC) cells. ZEB2-induced EMT enhances the expression of Excision Repair Cross-Complementation group 1 (ERCC1) and other materials related to the nucleotide excision repair (NER) pathway, leading to resistance to oxaliplatin (33). *In vitro* studies have shown that the deficiency of FBXW7 desensitizes the CRC cells to 5-fluorouracil (5-FU) and oxaliplatin chemotherapeutics induced by ZEB2/EMT (34).

### 2.2 FBXW7 regulates drug resistance by degrading cell cycle regulators

p53 acts as a tumor suppressor gene, inducing the cell cycle to arrest at a particular period and enhancing cell apoptosis. As a substrate of FBXW7, p53 can be degraded after ubiquitination (35). Both *in vitro* and *in vivo* assessments have indicated that in CRC, the reduction of FBXW7 promotes cells to have a higher tolerance to oxaliplatin by the abnormal transcription of phosphorylated p53 at Serine 15 (36). p53 has been implicated in many contexts in relation to tumor drug resistance. It has been shown to contribute to the acquisition of resistance to treatment with targeted MAPK inhibitors (37). For example, inhibition of p53 blocks the slow-cycling phenotype and sensitizes melanoma cells to BRAF/MEK inhibition (38). In addition, p53 ubiquitination can upregulate IL5RA and ultimately accelerate CDDP resistance in uveal melanoma (39). What’s more, p53 contributes to the induction of EMT and drug resistance through direct interaction with PRP4 and actions to promote the upregulation of HIF-1α and miR-210 (40).

Cryptochrome 2 (CRY2), a significant circadian clock protein related to the cell cycle, is reported to have a higher expression in CRC, which could be reversed by FBXW7. Overexpression of FBXW7 enhances the sensitivity of CRC to oxaliplatin along with the descending of CRY2 (41). The interaction between FBXW7 and CRY2 is regulated by metastasis-associated lung adenocarcinoma (LUAD) transcript 1 (MALAT1) (42).

Cyclin E is essential for the normal functions of the cell cycle, especially in G1 and S phases. FBXW7 degradation in CRC induces the stability of Cyclin E, which is regulated by Polo-like kinase 2 (PLK2) and RPTOR-independent companion of mTOR complex 2 (RICTOR) (43, 44). Increased levels of Cyclin E1 lead to drug resistance in many cancer cells, including ovarian cancer that is resistant to the standard platinum-taxane chemotherapy, the human epidermal growth factor receptor -2 (HER2) positive breast cancers resistant to trastuzumab and breast cancer resistant to cyclin-dependent kinase 4/6 (CDK 4/6)-inhibitors (45). In HER2^+^ breast cancer, overexpression of Cyclin E causes increased non-classical phosphorylation of SMAD Family Member 3 (SMAD3), conferring trastuzumab resistance (46).

### 2.3 FBXW7 regulates drug resistance by degrading proteins of Notch-signaling pathways

Notch signaling regulates the cell differentiation decisions (47). The decreased levels of FBXW7 cause the accumulation of Notch1 in cholangiocarcinoma (48). Overexpression of Notch1 intracellular domain (NICD) is also observed in the primary chronic lymphocytic leukemia (CLL) cells activated by FBXW7 mutation (49). Similarly, mice with FBXW7 mutation show haploinsufficiency for Notch degradation (47). Once FBXW7 is inhibited, Notch pathway can be activated, as well (50). High expression of Notch1 is associated with the EMT in ovarian cancer tissue, conferring chemo-resistance on ovarian cancer cells (51). Notch activity is associated with both tumor immune evasion and tumor resistance to therapy. The loss of Notch activity in glioma mouse models impairs major histocompatibility complex (MHC-I) and cytokine expression and inhibits the recruitment of anti-tumor immune cell populations. In addition, Notch-depleted glioma cells develop resistance to interferon-g and TAMs re-education therapy (52).

### 2.4 FBXW7 regulates drug resistance by degrading proteins of mTOR -signaling pathways

mTOR, the mammalian target of rapamycin, regulates protein synthesis. In many cancers, the mTOR signaling pathway is activated, which plays crucial parts in anti-apoptotic to trigger drug resistance (53). The activation of the mTOR pathway results in drug resistance in many cancers, inducing breast cancer resisted to trastuzumab, tamoxifen and endocrine therapy, prostate cancer resisted to vincristine, and ovarian cancer resisted to cisplatin (53). FBXW7 targets mTOR and mediates mTOR degradation in the tumor through direct physical combination with mTOR (54). On the other hand, the inhibition of FBXW7 expression activates the mTOR signaling pathway (55). mTOR pathway plays a central role in the regulation of protein synthesis, ribosomal protein translation and cap-dependent translation. In particular, it plays a central role in mediating mRNA translation of proteins associated with cell cycle progression. Dysregulation of mTOR signaling is frequently associated with tumorigenesis, angiogenesis, tumor growth and metastasis (53). For example, by activating the PI3K/AKT/mTOR signaling pathway, C2orf40 inhibits metastasis of nasopharyngeal carcinoma cell and regulates chemoresistance and radiation resistance (56). FXYD5 enhances resistance of HCC cells to sorafenib (57) and lncRNA enhances resistance to 5-fluorouracil in gastric cancer cells (58). In addition to these treatments, the mTOR pathway also affects immunotherapy. Experimental evidence suggests that modulation of mTOR signaling can alter the response to immune-checkpoint inhibitors (59). mTOR also has an effect on the differentiation of memory T cells. mTOR inhibition has been shown to favor the generation of CD8^+^ memory T cells (60).

### 2.5 FBXW7 regulates drug resistance by degrading antiapoptotic protein

MCL-1 is member of the B cell lymphoma 2 (Bcl-2) family that plays a key role in the regulation of cellular apoptosis. In colon cancer cells, FBXW7 is a critical component of regorafenib therapy. Regorafenib treatment requires phosphorylation and ubiquitination of MCL. Specifically, GSK3 phosphorylates MCL-1, which triggers its ubiquitination by the wild-type FBXW7-MCL-1 complex (61). In FBXW7-deficient T-ALL cell lines, overexpression of MCL-1 is observed, leading to the resistance of ABT-737, a Bcl-2 antagonist (62). However, upregulation of MCL-1 expression in FBXW7-deficient T-ALL cells increases sensitivity of ABT-737, suggesting that the deletion of FBXW7 causes the drug resistance to Bcl-2 antagonist in T-ALL cell lines through an MCL-1 mediated pathway (62). Similarly, lack of FBXW7 expression induces upregulation of MCL-1 in cholangiocarcinoma, resulting in resistance to cisplatin (48). Mcl-1 inhibitors can overcome intrinsic and acquired regorafenib resistance in CRCs by restoring the apoptotic response. The same conclusion can be obtained in CRCs with FBXW7 mutations (63).

## 3 FBXW7 is affected by upstream regulation

The expression of FBXW7 which regulates targeted proteins through ubiquitination, is itself regulated by other molecules (64). These upstream effects can be divided into three categories: the effect on FBXW7 transcription, the effect on FBXW7 translation, and the effect on FBXW7 protein stability (**Supplementary Table 2**).

### 3.1 The effects on FBXW7 transcription

#### 3.1.1 Methylases regulate FBXW7 transcription

In sunitinib-resistant cells, the SET domain containing 2 (SETD2) is downregulated compared with sensitive cells. The H3K36me3 marks are present in the *FBXW7 gene* allowing SETD2 to achieve epigenetic regulation of *FBXW7 DNA* through H3K36me3 (27). In pancreatic cancer, deletion of SETD2 leads to decreased expression of FBXW7, increasing the accumulation of MYC, an FBXW7 substrate, without significantly altering other FBXW7 substrates, such as mTOR and Cyclin E (65).

As a type II arginine methyltransferase, protein arginine methyltransferase 5 (PRMT5) establishes repressive histone makers to silence the target gene including FBXW7 (66). PRMT5’s oncogenic features include the inhibition of anti-tumor genes such as *E-cadherin* and *FBXW7* (66). Structurally, PHD finger protein 1 (PHF1), PRMT5-WDR77 and cullin4B-ring E3 ligase complex (CRL4B) act as a complex, occupying the *FBXW7* promoter. Functionally, the downregulation of PHF1, PRMT5, or CRL4B significantly reduces the corresponding proteins’ binding level to increase FBXW7 expression at the transcriptional and protein levels (66).

#### 3.1.2 Transcription factors regulate FBXW7 transcription

CCAAT/enhancer-binding protein-δ(C/EBPδ) binds to DNA *via* leucine zipper scissors and participates in transcriptional regulation. In addition to its role in cell apoptosis, it directly suppresses the expression of FBXW7, leading to subsequent effects such as the stability of mTOR and activation of the Notch pathway (50, 67).

Hairy enhancer split (Hes5), a downstream effector of the Notch1 pathway, negatively regulates FBXW7 as a transcriptional repressor. Studies on mice have shown that deficiency of Hes5 leads to the restored induction of Fbxw7 haploinsufficiency (47). A positive feedback loop consisting of NICD/Hes5/FBXW7 was identified in colon cancer cells, where the negative regulation of FBXW7 by Hes5, particularly its repressive effect on the transcription of FBXW7, is extremely significant (47).

Forkhead box A1 (FOXA1) also binds to the promoter of *FBXW7* in sunitinib-resistant renal cells, facilitating its expression. The downregulation of FBXW7 induced by FOXA1 reduction is responsible for the sunitinib-resistant in renal cancer (35).

### 3.2 The effects on FBXW7 translation

#### 3.2.1 miRNAs inhibit FBXW7 translation

Of the 69 F-box proteins, only a dozen are regulated by non-coding RNA, including FBXW7 (68). Although different miRNAs play a role in different tumors, they all generally inhibit the translation of FBXW7, resulting in decreased FBXW7 protein expression and indirect drug resistance (**Supplementary Table 3**). Most miRNAs repress FBXW7 expression by binding to the 3’-untranslated region (3’-UTR) of FBXW7 mRNA. Inhibition of FBXW7 by miRNA weakens its regulatory effect on downstream substances, affecting the sensitivity of each tumor to drugs.

Exosome transfer of miR-25-3p regulates glioblastoma (GBM) cell proliferation and Temozolomide (TMZ) resistance by inhibiting FBXW7 and subsequently promoting the expression of oncoproteins such as c-MYC and Cyclin E. Overexpression of miR-25-3p significantly inhibits wild type FBXW7, while knockdown of miR-25-3p significantly increases wild type FBXW7 in GBM cells. For FBXW7 with binding site mutation, the effect induced by miR-25-3p overexpression or knockdown can be eliminated (69). In addition to GBM, miR-25 also inhibits FBXW7, increasing the resistance of hepatocellular carcinoma to sorafenib (70).

Overexpression of miR-27b-3p mimics in myeloma fibroblasts significantly inhibits FBXW7 mRNAs and protein. On the contrary, forced expression of miR-27b-3p inhibitors in myeloma fibroblasts inhibits the level of miR-27b-3p and induces a significant increase in the concomitant increase of FBXW7 mRNAs and protein. Based on these results, FBXW7 is the target mRNA of miR-27b-3p (71).

Clinical data shows that the expression of miR-32 is increased in multiple myeloma (MM) tissues, and cells with high expression of miR-32 are often accompanied by low expression of FBXW7. In addition, *in vitro* experiments also found that miR-32 transfected cells have lower expression of FBXW7 and higher expression of cancer-related proteins c-Jun and c-MYC. On the other hand, anti-miR-32 transfected cells show higher FBXW7 expression and lower c-Jun and c-MYC expression. These studies indicate that the expression of miR-32 was negatively correlated with the expression of FBXW7 mRNA (72). A similar phenomenon is observed in breast cancer cells as well, where miR-32 is often overexpressed, inhibits cell apoptosis and promotes proliferation and migration. MiR-32 binds to the 3’-UTR of FBXW7, suggesting that FBXW7 is a direct target of miR-32. Overexpression of miR-32 decreases the level of FBXW7 protein, which are also negatively correlated with the level of FBXW7 mRNA. Comparing the FBXW7 mRNA 3 ‘-UTR mutants with the FBXW7 mRNA 3’-UTR wild-type shows that miR-32 can down-regulate its expression by directly targeting the 3’-UTR of FBXW7. Even the FBXW7 3’-UTR mutant group shows decreased expression compared to the blank control (73).

The expression of miR-92a is significantly upregulated in cervical cancer (CC) tissues and cell lines, it inhibits the expression level of FBXW7 by directly binding to the 3’-UTR of FBXW7 mRNA. This results in a negative correlation between the expression of FBXW7 and the level of miR-92a in CC tissues. The overexpression of miR-92a can significantly promote the transition of the cell cycle from the G1 phase to the S phase and enhance the invasiveness of CC cells. However, FBXW7 can reverse the carcinogenic effect of miR-92a (74).

miR-92a-3p significantly suppresses FBXW7 with wild-type 3’ non-coding region but not in FBXW7 with a 3’ non-coding region mutation. In addition, the level of FBXW7 in CRC cells with miR-92a-3p was significantly decreased (75). The high expression of miR-92a-3p activates the Wnt/β-catenin pathway, which suppresses the mitochondrial apoptosis by directly inhibiting FBXW7, thereby promoting stem cell differentiation, endothelial cell metastasis, and 5-FU/oxaliplatin resistance in CRC (75).

In hepatocellular carcinoma (HCC), overexpression of miR-155-3p induces FBXW7 to be downregulated at both mRNA and protein levels. Specific inhibitors of miR-155-3p increase FBXW7. In terms of mechanism, the 3 ‘-UTR region of FBXW7 mRNA contains a binding site of miR-155-3p, which can directly bind to FBXW7 mRNA and regulate the expression of FBXW7 through translation inhibition (76).

miR-182 reduced the FBXW7 protein levels through targeting FBXW7 3’-UTR directly in breast cancer cells, but has no significant effect on mRNA levels. Conversely, inhibition of miR-182 increased the FBXW7 protein levels in human breast cancer cells (77). At the same time, miR-182 can only have an effect on wild-type FBXW7, but cannot change the mutant FBXW7 (78). The similar phenonenon can also be found in other cancers. In CC cells and renal cancer cells, miR-182-5p directly binds to the 3’-UTR of FBXW7 mRNA and inhibits the expression of wild-type FBXW7 proteins because FBXW7 contains a hypothesized binding site for miR-182-5p in its 3’-UTR (79, 80).

Studies have indicated that miR-223 targets FBXW7 and stimulates its degradation (81). The detection of luciferase activity report shows that miR223-3p can directly combine with the 3 ‘-UTR of FBXW7. MiR-223-3p partially plays its carcinogenic role by reducing the expression of FBXW7, thereby promoting the invasion and metastasis of breast cancer cells (55). The inhibitory effect of miR-223 on FBXW7 leads to the promotion of adriamycin resistance in GC cells (82). Also, miR-223-induced decrease of FBXW7 expression causes resistance to cisplatin in non-small cell lung cancer (83). In CRC, miR-223 promoted adriamycin resistance in cancer cells by inhibiting FBXW7 (84).

MiR-363 was found to reduce the expression of FBXW7 in GC cells with wild-type 3’-UTR, but it had no effect on the expression of transfected mutant FBXW7 3’-UTR. Moreover, the overexpression of miR-363 was associated with a decrease in the mRNA expression of FBXW7 in GC cells, indicating that miR-363 negatively regulates FBXW7 mRNA levels in GC tissues (85).

Studies in HCC cells indicate that miR-367 can directly target FBXW7 and negatively regulate FBXW7 expression in HCC cells (86). Studies in non-small cell lung cancer have also reported FBXW7 as a downstream target of miR-367 (87). Co-transfection of miR-367 mimics with luciferase reporter structure containing FBXW7 3’-UTR showed that the cellular luciferase activity of transfected miR-367 mimics was down-regulated, suggesting that miR-367 regulates its expression by directly targeting the 3’-UTR of FBXW7 (87).

In HeLa cells, FBXW7 is inhibited by miR-586 after transcription. The presence of miR-586 mimics significantly inhibited the level of FBXW7, leading to a decrease in endogenous FBXW7 protein and mRNA levels. Conversely, the miR-586 inhibitors increase the FBXW7 expression. However, when 3’-UTR of FBXW7 is mutated, miR-586 has largely no effect (88).

Exosomes of the cisplatin resistant GC cells have also been found to enhance cisplatin resistance by targeting FBXW7 with miR-500a-3p *in vitro* and *in vivo*, but FBXW7 can rescue cisplatin resistance by inhibiting cancer stem cells properties. In mice with abdominal tumorigenesis of the GC cells, the reintroduction of FBXW7 was observed to inhibit tumor growth and metastasis under cisplatin treatment (89). Finally, decreased expression of miR-5000-3p inhibited the proliferation and migration of laryngeal cancer cells, while upregulation of MIR22HG expression led to an increase in FBXW7 expression and protein level. Luciferase reporting experiments also demonstrated that upregulation of miR-5000-3p can reduce wild type FBXW7 reduction, but it also has no significant effect on FBXW7 mutation (90).

#### 3.2.2 lncRNAs enhance FBXW7 translation

In contrast to miRNA, long-non-coding RNA (lncRNA) plays a facilitative role for FBXW7. In general, lncRNA acts as a molecular sponge in cells, reducing the binding of miRNA and FBXW7 mRNA by competitively binding to the sites on FBXW7 mRN A, thereby reducing the inhibitory effect of miRNA on FBXW7. This inhibition of miRNAs results in an increase in FBXW7 expression, which leads to the degradation of the corresponding substrate and an increase in tumor sensitivity to drugs. However, the specific lncRNAs that play a role in different tumors vary. **Supplementary Table 4** illustrates the corresponding relationships (**Supplementary Table 4**).

In laryngeal cancer cells, the high expression of lncRNA-MIR22HG inhibits proliferation and migration, but its expression is down-regulated. LncRNA-MIR22HG regulates the expression of FBXW7 in the laryngeal cancer cells by competitively binding of miR-5000-3p, erasing its expression. E2F6 has been found to inhibit the transcription of lncRNA-MIR22HG in the laryngeal cancer cells (90).

LncRNA-MT1JP is significantly lower in the GC tissues than in the adjacent normal tissues, and the increase of lncRNA-MT1JP is significantly correlated with lymph node metastasis and advanced stage. Overexpression of lncRNA-MT1JP inhibits cell proliferation, migration, and invasion, promoted cell apoptosis *in vitro*, and inhibits tumor growth and metastasis *in vivo*. Therefore, the GC patients with a high expression of lncRNA-MT1JP have a better survival rate. lncRNA-MT1JP regulates the expression of FBXW7 by competitively binding to miR-92a-3p.The RNA level of FBXW7 was significantly increased in the overexpressed lncRNA-MT1JP cells (91).

LncRNA-MIF induction increases FBXW7 mRNA and protein levels. These data suggest that lncRNA-MIF may shorten the half-life of c-MYC by increasing the expression of Fbxw 7. There is no direct correlation between lncRNA-MIF and FBXW7, but lncRNA-MIF can act as a microRNA sponge to regulate the expression of FBXW7. LncRNA-MIF specifically interacts with miR-586 in the cytoplasm to competitively bind miR-586 and reduces its inhibitory effect on FBXW7. Therefore, lncRNA-MIF can indirectly upregulate FBXW7 (88).

Lnc-CASC2 expression is significantly down-regulated in HCC tissues, especially in invasive and recurrent cases. *In vitro* and *in vivo* experiments demonstrate that CASC2 can inhibit the migration and invasion of HCC cells by down-regulating the EMT process. CASC2 competitively binds miR-367 in the HCC cells by functioning as a molecular sponge. In conclusion, CASC2 positively regulates the expression of FBXW7 by sponge miR-367 (86).

Lnc-LINC00173 acts as a molecular sponge of miR-182-5p and reverse-regulates the level of miR-182-5p in the CC cells. Overexpression of lnc-LINC00173 decreases the level of miR-182-5p in HeLa cells, while downregulation of lnc-LIN00173 increases the expression of miR-182-5p in the CC cells. Since FBXW7 is the target of miR-182-5p, lnc-LINC00173 positively regulates the expression of FBXW7 by inhibiting miR182-5p in the CC cells, thus inhibiting the cell proliferation (79).

#### 3.2.3 METTL3 and m6A -mediated FBXW7 RNA modification enhance FBXW7 translation

METTL3 is a “writer” enzyme that is part of the m^6^A methyltransferase complex. In LUAD, the high expression of METTL3 induces the m^6^A modification in FBXW7 mRNA, rescuing the levels of FBXW7 protein expression (92). The m^6^A methylation modification site is present in FBXW7 mRNA, and the expression of METTL3 and FBXW7 is correlated. In LUAD, overexpression of METTL3 promotes m^6^A modification, which enhances the translation of FBXW7. *In vivo* experiments have also demonstrated that overexpression of FBXW7 can rescue the anti-tumor effects attenuated by METTL3 knockdown (92).

#### 3.2.4 Piwil1 inhibits FBXW7 translation by reducing mRNA stability

Piwi Like RNA-Mediated Gene Silencing 1 (Piwil1), a member of the argonaute proteins subfamily, is highly expressed in GBM. Knock out of Piwil1 increases the level of FBXW7, as Piwil1 negatively regulates the stability of FBXW7 mRNA. However, Piwil1 regulates FBXW7 through an unknown mechanism, rather than directly binding to FBXW7 mRNA (93).

### 3.3 The effects on FBXW7 protein

#### 3.3.1 PLK1 and ERK1/2 suppress the level of FBXW7 by degrading FBXW7 protein

Polo-like kinase 1 (PLK1) participates in various stages of cell division. In medulloblastoma, the suppression of PLK1 promotes the stability of FBXW7 by downregulation of FBXW7 poly-ubiquitination and degradation, indicating that PLK1 has a negative effect on FBXW7 (17). The PLK1 inhibitor BI6727 stabilizes the FBXW7 protein in Burkitt lymphoma cells, further supporting its negative regulatory effect on FBXW7 (94).

In drug resistance cells, high expression of ERK1/2 induces FBXW7 degradation (25). *In vivo* studies have revealed that downregulation of ERK1/2 reverses the expression of FBXW7 at Ser303/307 in the mouse embryonic fibroblasts (25).

The thyroid hormone receptor interactor 12 (TRIP12), an E3 ubiquitin ligase of the HECT domain, is a negative regulator of FBXW7 stability. Knockdown of TRIP12 does not affect FBXW7 mRNA levels but increases the amount of endogenous FBXW7α protein. In contrast, increased proteasomal degradation of FBXW7 protein accumulation and secondary FBXW7 substrate MCL-1 due to TRIP12 inactivation sensitizes cancer cells to antitubulin chemotherapy (95).

TRP120 ligase, a functional HECT E3 ligase, is a substrate for the tumor suppressor FBXW7. TRP120 ligase maintains the stability of Notch and other tumor proteins involved in cell survival and apoptosis by degrading FBXW7, thereby downregulating the innate immune host defense system and promoting ehrlichiosis (96).

## 4 Immunity therapy

### 4.1 FBXW7 and the immune system in cancer cells

FBXW7 plays a role in regulating the immune system in cancer cells. Eyes absent homolog 2 (EYA2) is degraded by FBXW7-mediated ubiquitination. Downregulation of EYA2 leads to weak mesenchymal phenotypes, increased immunogenicity of cancer cells, reduced carcinogenicity, including tumor growth and metastasis, and increasing infiltration level of natural killer cells (NK cells) and cytotoxic T cells (97).

FBXW7 also influences macrophages, as its deficiency leads to improved expression of chemokine C-C Motif Chemokine Ligand 2 (CCL2) in serum, resulting in the recruitment of macrophages and monocytic myeloid-derived cells and eventually resulting in tumor metastasis (8). Additionally, FBXW7 regulates macrophages through an mTOR-related pathway. The downregulation of FBXW7, inhibited by calcium/calmodulin-dependent protein kinase IV (CaMKIV), leads to increased mTOR in macrophages, which results in the Lipopolysaccharide (LPS)-induced mediation of macrophages subsequently (98).

The lack of FBXW7 promotes cell proliferation of double-positive T-cells due to the accumulation of c-MYC, but it does not make an obvious change in single-positive T-cells (99).

Furthermore, FBXW7 is highly expressed in the germinal center of B and B1 cells, where it maintains the homeostasis of mature B cells and B-1 cells. It also plays a critical role in BCR-mediated B cell proliferation and survival. The absence of FBXW7 in B cells impairs the Ig switching and the germinal center function, including switching recombination and the affinity maturation of antibodies, resulting in a weakened memory antibody response. This effect may be due to the effect of FBXW7 on B-cell lymphoma 6 (BCL6), an essential protein that initiates and maintains the germinal center response. FBXW7 provides a potential treatment for GC-related and autoantibody-induced autoimmune diseases (100).

### 4.2 FBXW7 and immunotherapy

#### 4.2.1 FBXW7 and PD-L1 protein

FBXW7 regulates the PD-L1 protein indirectly. In tumors where FBXW7 is silenced, the expression of PD-L1 is significantly increased through the transcriptional regulation of signal transducer and activator of transcription 3 (STAT3) (101).

Once the activity of FBXW7 is inhibited, c-MYC protein accumulates, resulting in the up-regulation of PD-L1. Therefore, the expression level or mutation status of FBXW7 and c-MYC proteins may also reflect the efficacy of combined therapy (102).

The loss of function (mutation or deletion) of tumor suppressor gene FBXW7 and the overexpression of c-MYC oncoprotein are related to the expression level of PD-L1 and poor prognosis in some malignant tumors (102).

#### 4.2.2 FBXW7 and immunotherapy outcomes

FBXW7 has been shown to play a crucial role in promoting anti-tumor immunity (97). The inactivation of FBXW7 has been associated with the development of resistance to the PD-1 blockade in immunoreactive animals, likely due to alterations in the immune microenvironment. Specifically, FBXW7 has been linked to the downregulation of type I interferon induction and major MHC-I expression, as well as the decreased expression of dsRNA sensors such as melanoma differentiation-associated protein 5 (MDA 5) and retinoic acid-inducible gene I (RIG-I) (103). Conversely, FBXW7 can enhance PD-1 blockade therapy by promoting the degradation of EYA2. Targeting EYA2 tyrosine phosphatase activity in mice with tumors exhibiting a reduced mesenchymal phenotype has been shown to enhance cancer cell immunogenicity, resulting in suppressed tumor growth and improved anti-PD-1 therapeutic response (97). The restoration of dsRNA perception in FBXW7 deficient cells was sufficient to sensitize them against anti-PD-1 (103). For example, overexpression of mitochondrial antiviral-signalling protein(Mavs) makes the FBXW7 deficient tumor sensitive to PD-1 blocking, delays the growth of Fbxw7-deficient tumors and prolongs the survival of anti-PD-1-treated Fbxw7-deficient tumor-carrying mice (103).

Taken together, these findings suggest that screening for FBXW7 status could be a valuable predictor of clinical response to anti-PD-1 immunotherapy, and that targeting FBXW7 may be a promising strategy for enhancing anti-tumor immunity (104). Dysfunctional FBXW7 is implicated in defective antigenic peptides formation, tumor development, and malignant tumor manifestations, all of which increases resistance to PD-1 therapy in melanoma. In contrast, reactivation of FBXW7 can improve response to PD-1 blockade (103).

**Figure 4:**
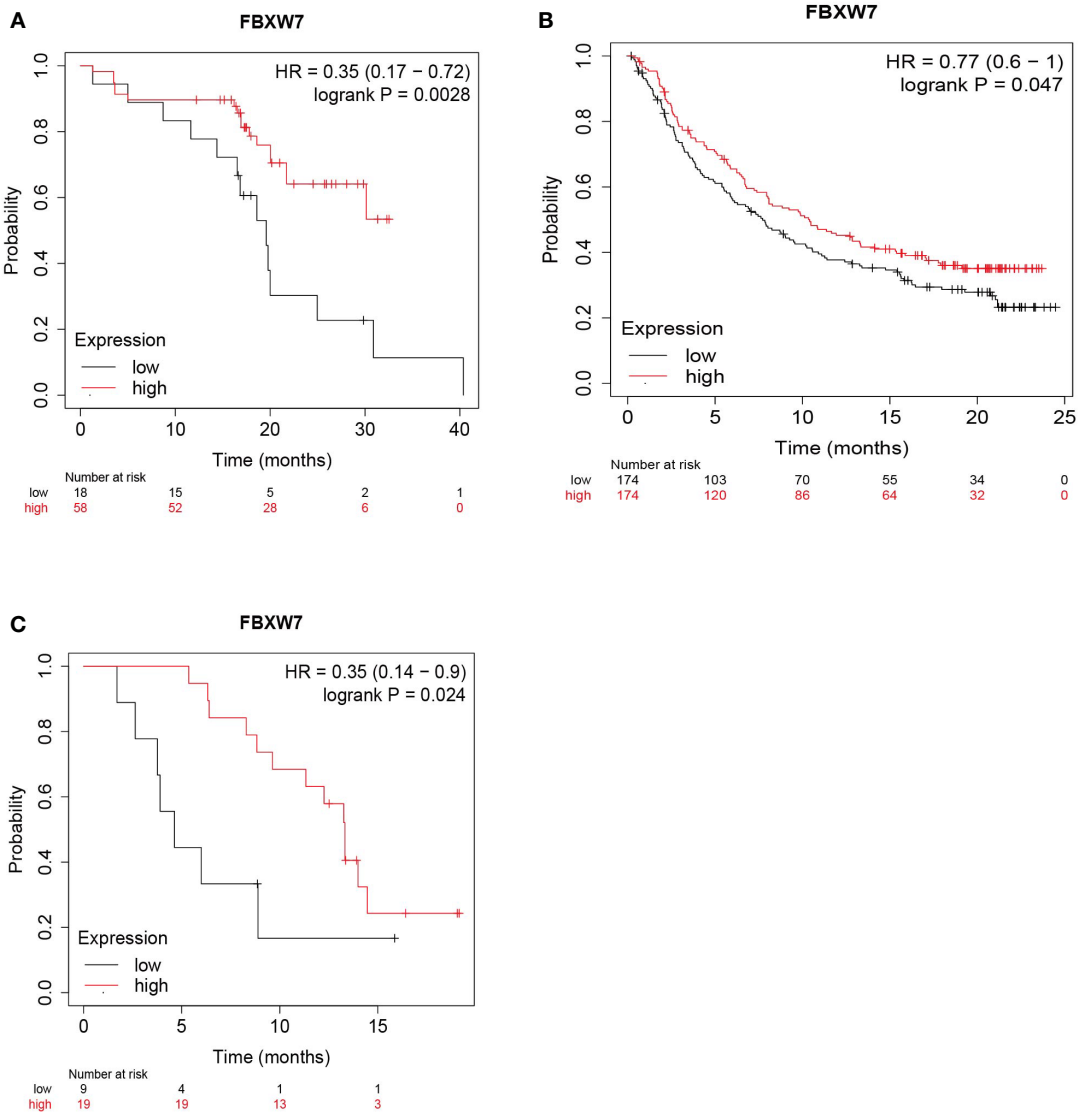
The survival rate of patients with high and low expression is performed using Kaplan Meier plotter. (https://kmplot.com/analysis/index.php?p=service) **(A)** Esophageal adenocarcinoma patients who accept all anti-PD-L1 treatment shows that people with high levels of FBXW7 owns a longer survival time. **(B)** Urothelial carcinoma patients who accept all anti-PD-L1 treatment shows that people with high levels of FBXW7 owns a longer survival time. **(C)** Glioblastoma patients who accept all anti-PD-1 treatment shows that people with high levels of FBXW7 owns a longer survival time.

The efficacy of anti-PD-L1/PD-1 immunotherapy is known to be influenced by the level of PD-L1 protein associated with tumor cells (105). Interestingly, data from online databases show that esophageal adenocarcinoma and urothelial carcinoma patients with high expression of FBXW7 exhibit longer survival times following anti-PD-L1 therapy compared to those with low FBXW7 expression (**Figures 4A, B**). A similar trend is observed in GBM patients who are treated with anti-PD-1 therapy (**Figure 4C**). These results suggest that FBXW7 expression is a potential prognostic indicator for the response to immunotherapy in cancer patients.

### 4.3 Other clinical therapy

Although the loss of FBXW7 makes tumors resistant to many drugs, there are treatments that work on cells lacking FBXW7. For instance, Lycorine hydrochloride (LH) attenuates the level of MCL-1 by upregulating FBXW7 in the GC cells. The cell cycle stops at the S phase as a result of insufficient MCL-1, followed by the BCL2-drug-resistant GC cells apoptosis. LH has been reported to have a positive effect on inhibiting cells growth and tumorigenesis (106).

Aside from increasing FBXW7 expression, other drugs such as tigecycline and histone deacetylase (HDAC) inhibitors can target the Fbxw7-deficient cells and reverse the harm caused by the reduction of FBXW7 in humans. Tigecycline, for instance, has been shown to be toxic to FBXW7 knockout cells in the heterograft colorectal adenocarcinoma cells. tumor cells that lack FBXW7 show no response to paclitaxel, but this effect is attenuated by tigecycline. Other drugs, such as tigecycline or oligomycin, which activate the integrated stress response (ISR), are also toxic to FBXW7-deficient cells (107). Entinostat MS-275, a Class 1 HDAC inhibitor, has been found to be highly effective in treating Fbxw7-deficient NSCLC cells. MS-275 therapy has been shown to overcome paclitaxel resistance, suggesting that HDAC inhibitors may have a therapeutic potential in treating FBXW7- deficient NSCLC with invasive paclitaxel-resistant (15).

## 5 Other F-box proteins regulate drug resistance in cancer

F-box proteins play a pivotal role in the progression and development of various human malignancies by regulating the turnover of critical factors involved in a range of cellular processes (108). Apart from FBXW7, other F-box proteins also impact tumor drug resistance by regulating the corresponding substrate proteins (**Supplementary Table 5**).

High mRNA expression of FBXO 4 is significantly associated with better survival in luminal B-type BC patients (109). In patients with stage III breast cancer, high expression of FBXWO4 and FBXL3 mRNA is associated with better survival. FBXO4 regulates the therapeutic outcome in metastatic breast cancer, by contributing to the stability of intercellular adhesion molecule-1 (ICAM-1) (110). In head and neck squamous cell carcinoma, the reduction of FBXO 4 and its substrate Fxr1 correspondingly to the suppression of tumorigenesis (111).

FBXO5, also known as early mitosis Inhibitor 1(EMI1), is involved in cell sensitivity to poly ADP-ribose polymerase (PARP) inhibitors by targeting RAD51 degradation in breast cancer (112).

FBXO6 controls the degradation of checkpoint kinase 1 (Chk1) and confers the sensitivity to certain anticancer drugs, including camptothecin, in tumor cells, such as non-small cell lung cancer, GBM, and breast cancer (113). FBXO6 also inhibits cell proliferation, induces apoptosis, and enhances cell sensitivity to cisplatin by Chk1 in non-small cell lung cancer (114).

BCL-2 upregulation is one of the causes of ibrutinib resistance in mantle cell lymphoma (115). However, FBXWO10 targets BCL-2 and induces its degradation (116). Therefore, upregulating FBXO10 may be an effective way to overcome resistance to ibrutinib in MCL (117).

CD147 (Basigin), a transmembrane glycoprotein of the immunoglobulin superfamily, is associated with chemotherapy resistance in various human malignancies (118). FBXO22 mediates the polyubiquitination and degradation of CD147 by recognizing CD147-ICD, causing a reversal of cisplatin resistance in cancer cells (119).

Deletion of FBXL5 and B-cell translocation gene 3(BTG3) increases cell invasion and cisplatin resistance in CC (120). In GC cells, loss of FBXL5 increased cisplatin resistance by activating ERK and p38. Sufficient FBXL5 combined with Rho GDP dissociative inhibitor β(RhoGDI2) reduced the cisplatin resistance of RhogDi2-mediated GC cells (121). FBXL5 targets ubiquitination and degradation of human single-stranded DNA binding protein 1(HSSB1), leading to ATM activation and enhanced radiosensitivity and chemotherapy sensitivity in lung cancer (122).

The down-regulation of FBXL7 inhibits the SURVIVIN degradation, leading to increased the SURVIVIN protein levels and increased drug resistance (123). The down-regulation of the FBXL7 expression also increases the sensitivity of ovarian cancer cells to paclitaxel (124).

## 6 Conclusion

In conclusion, FBXW7 has been shown to be associated with patient survival and prognosis with higher FBXW7 linked to longer disease-free and overall survival. Of particular interest is its impact on drug resistance in tumors. FBXW7 targets the degradation of various molecules, including MCL-1, NFAT1, p53, CRY2, c-MYC, ZEB2, Snail1, pHSF1, Notch1, PD-1, Cyclin E, mTOR, and c-Jun to regulate the corresponding pathway and ultimately reduce drug resistance. Other molecules, such as FOXA1, SETD2, Hes5, C/EBP-δ, PRMT5, METTL3, Piwil1, PLK1, ERK1/2, and a series of miRNA, indirectly modulate the sensitivity of cancer cells to drugs by regulating FBXW7.

Moreover, FBXW7 has an impact on the immune microenvironment and it can affect the efficacy of immunotherapy by amplifying the therapeutic effect of anti-PD-L1. For the FBXW7-deficiency cells, certain drugs have shown therapeutic potential. However, further and more extensive experiments are necessary to fully understand the impact FBXW7 reduction and mutation in cells.

## Author contributions

SC, JZ, JCL, QL wrote the original draft. LM, QW, RM and JL contributed to the final draft. All authors contributed to manuscript revision, read, and approved the submitted version.
