# Supplementary material for: FBXW7 attenuates tumor drug resistance and enhances the efficacy of immunotherapy
Source: Front Oncol. 2023 Mar 14;13:1147239. doi: 10.3389/fonc.2023.1147239 (PMC10043335; doi:10.3389/fonc.2023.1147239)
Supplement: Supplementary file 1 [file Table_1.docx]

Supplementary Material

FBXW7 reduces tumor resistance and improve the efficacy of immunotherapy

Shimin Chen^1,2^, Jiaojiao Zhao^1,2^, Jichun Lin^1,2^, Qian Lin^1,2^, Jia Liu^1^, Qiang Wang^1^, Leina Ma^1*^

**Correspondence:** Leina Ma: leinama@gmail.com

# Supplementary Tables

Supplementary Table1: This table describes how FBXW7 regulates the drug sensitivity by degrading the corresponding substrates in various cancers. The drugs and therapy that can be affected by FBXW7 are indicated in the first column. The cancer type and the related substrates of FBXW7 are listed in the second and third columns. The fourth column enumerates the way that how FBXW7 regulates the drug sensitivity and how the FBXW7 mutation or deficiency leads to the drug resistance.

| drug | cancer | substrate | resistance mechanism | reference |
| --- | --- | --- | --- | --- |
| oxaliplatin | colorectal cancer | p53 | FBXW7 degrades P53 family of transcription factors and makes colorectal cancer cells sensitize to oxaliplatin | (28) |
| oxaliplatin | colorectal cancer | CRY2 | FBXW7 degrades CRY2 and makes colorectal cancer cells sensitize to oxaliplatin | (29) |
| oxaliplatin | colorectal cancer | ZEB2 | FBXW7 degrades ZEB2, inhibites EMT and makes colorectal cancer cells sensitized to oxaliplatin | (26) |
| cisplatin | non-small cell lung cancer | Snail | FBXW7 degrade Snai1 directly and inhibits EMT progress, increasing cisplatin sensitivity | (22) |
| cisplatin | ovarian cancer | Notch1 | FBXW7 degrades Notch1 and inhibits EMT, increasing cisplatin sensitivity | (38) |
| cisplatin | ovarian cancer | mTOR | FBXW7 degrades mTOR and makes ovarian cancer cells sensitize to cisplatin | (39) |
| cisplatin | cholangiocarcinoma | MCL-1 | FBXW7 degrades MCL-1, increasing cisplatin sensitivity | (35) |
| cisplatin | non-small cell lung cancer | Snail | FBXW7 degrade Snai1 directly and inhibits EMT progress, increasing cisplatin sensitivity | (22) |
| sorafenib | hepatoma | c-Jun | FBXW7 degrades c-Jun and promotes the sorafenib-mediated apoptosis | (17) |
| 5-FU | colorectal cancer | ZEB2 | FBXW7 degrades ZEB2, inhibits EMT and makes colorectal cancer cells sensitize to 5-FU chemotherapeutics | (26) |
| docetaxel, cisplatin,5-fluorouracil （TPF） | hypopharyngeal carcinoma | c-Jun | FBXW7 degrades c-Jun, restrains cell cycle progression and facilitates anti-apoptotic activity, thus relieving TPF resistance | (16) |
| paclitaxel | lung cancer | pHSF1 | FBXW7 degrades pHSF1 at Ser303/307, which reduces the transcription of MDR1 and suppresses drug resistance | (19) |
| standard platinum-taxane chemotherapy | ovarian cancers | cyclin E | FBXW7 degrades cyclin E and inhibits drug resistance | (33) |
| trastuzumab | breast cancers | cyclin E | FBXW7 degrades cyclin E and inhibits drug resistance | (33) |
| BH3-mimetics | tumor cells | cyclin E | FBXW7 degrades cyclin E and inhibits drug resistance | (33) |
| palbociclib | breast cancer | cyclin E | FBXW7 degrades cyclin E and inhibits drug resistance | (33) |
| Trastuzumab, Endocrine therapy, Tamoxifen | breast cancer | mTOR | FBXW7 degrades mTOR and inhibites mTOR signaling pathway, which inhibits drug resistance | (39) |
| vincristine | prostate cancer | mTOR | FBXW7 degrades mTOR and inhibits mTOR signaling pathway, which inhibits vincristine resistance | (39) |
| regorafenib | colon cancer | MCL-1 | FBXW7 degrades MCL-1, inhibits regorafenib resistance | (42) |
| ABT-737 | T-all cell lines | MCL-1 | FBXW7 degrades MCL-1, and suppresses drug resistance to Bcl-2 antagonist | (43) |
| sunitinib | renal cancer | NFAT1 | FBXW7 degrades NFAT1 inhibits sunitinib resistance | (20) |
| gamma-secretase inhibitor | T-all cell line | c-MYC | FBW7 degrades c-MYC and suppresses the resistance to γ-secretase inhibition | (15) |

Supplementary Table2：The upstream of FBXW7. The first column shows the effect level in which the corresponding upstreams regulate. The upstream molecules that regulate FBXW7 and their effects are indicated in the second and third columns. The processes of these upstream molecules are listed in the fourth column.

| effect level | upstream | effect to FBXW7 | | pathway | | reference | |  |  |  |
| --- | --- | --- | --- | --- | --- | --- | --- | --- | --- | --- |
| transcription of FBXW7 | SETD2 | Induces upregulation of FBXW7 | | SETD2 induces H3K36 trimethylation and enhance the *FBXW7* transcription | | (20) | |  |  |  |
| transcription of FBXW7 | | PHF1/PRMT5/CRL4B complex | | Induces downregulation of FBXW7 | | PHF1/PRMT5/CRL4B co-occupies the promoters of *FBXW7* and suppresses transcription | (45) | | |  |
| transcription of FBXW7 | C/EBP-δ | Induces downregulation of FBXW7 | | C/EBP-δ combines with *FBXW7* to inhibit transcription | | (37) | |  |  |  |
| transcription of FBXW7 | Hes5 | Induces downregulation of FBXW7 | | HES5 represses transcription of *FBXW7* | | (34) | |  |  |  |
| transcription of FBXW7 | FOXA1 | Induces upregulation of FBXW7 | | FOXA1 binds to FBXW7 promoter and suppresses transcription of *FBXW7* | | (20) | |  |  |  |
| translation of FBXW7 | | METTL3 | | Induces upregulation of FBXW7 | METTL3 increases m6A methylation modification and enhances FBXW7 translation. | | | (66) | | |
| Stability of FBXW7 mRNA | piwi | Induces downregulation of FBXW7 | | Piwil1 regulates FBXW7 mRNAs stability indirectly | | (67) | |  |  |  |
| FBXW7 protein | PLK1 | Induces downregulation of FBXW7 | | PLK1 increase FBXW7 auto poly-ubiquitination and proteasomal degradation | | (14) | |  |  |  |
| FBXW7 protein | ERK1/2 | Induces downregulation of FBXW7 | | ERK1/2 induces degradation of FBXW7 | | (19) | |  |  |  |

Supplementary Table3：The miRNA inhibits the expression of FBXW7 through various ways. This table describes the various types of miRNA which play a significant role in restraining FBXW7 indifferent cancer. The miRNA and their related cancer are indicated in the first and second columns. The results and pathway of their effects on FBXW7 are listed in the third and fourth columns.

| miRNA | cancer | effect to FBXW7 | pathway | reference |
| --- | --- | --- | --- | --- |
| miR - 25 - 3p | glioblastoma | Induces downregulation of FBXW7 | miR-25-3p targets FBXW7 mRNA 3'UTR directly and inhibits the expression of FBXW7 protein | (47) |
| miR-27b-3p | bone marrow fibroblasts | Induces downregulation of FBXW7 | miR-32 was increased in tissues and inhibit FBXW7 expression, leading to higher level of Notch and ERK1/2 | (48) |
| miR-32 | multiple myeloma disease | Induces downregulation of FBXW7 | miR-32 was increased in tissues and inhibited FBXW7 expression, leading to higher level of c-MYC and c-Jun | (49) |
|  | breast cancer | Induces downregulation of FBXW7 | miR-32 targets FBXW7 mRNA 3'UTR directly and inhibits the expression of FBXW7 protein | (50) |
| miR-92a | cervical cancer | Induces downregulation of FBXW7 | miR-92a targets FBXW7 mRNA 3'UTR directly and inhibits the expression of FBXW7 protein | (51) |
| miR-92a-3p | colorectal cancer | Induces downregulation of FBXW7 | miR-92a-3p targets FBXW7 3'UTR directly and inhibits the expression of FBXW7 protein | (52) |
|  | colorectal cancer | Induces downregulation of FBXW7 | miR-92a-3p targets FBXW7 3'UTR directly and inhibits the expression of FBXW7 protein | (52) |
| miR-155-3p | hepatocellular carcinoma | Induces downregulation of FBXW7 | miR-155-3p targets FBXW7 mRNA 3'UTR directly and inhibits the expression of FBXW7 protein | (53) |
| miR-182 | breast cancer | Induces downregulation of FBXW7 | miR-182 targets FBXW7 3'UTR directly and inhibits the expression of FBXW7 protein | (54) |
| miR-182-5p | renal cancer | Induces downregulation of FBXW7 | miR-182-5p targets FBXW7 mRNA 3'UTR directly and inhibits the expression of FBXW7 protein | (57) |
|  | breast cancer | Induces downregulation of FBXW7 | miR-182-5p targets FBXW7 mRNA 3'UTR directly and inhibits the expression of FBXW8 protein | (96) |
|  | cervical cancer | Induces downregulation of FBXW7 | miR-182-5p targets FBXW7 mRNA 3'UTR directly and inhibits the expression of FBXW7 protein | (56) |
| miR-223 | breast cancer | Induces downregulation of FBXW7 | miR223-3p can be directly combined with the 3 '-UTR of FBXW7 and reduce its expression | (41) |
| miR-363 | gastric cancer | Induces downregulation of FBXW7 | miR-363 targets FBXW7 mRNA 3'UTR directly and inhibits the expression of FBXW7 protein | (59) |
| miR-367 | hepatocellular carcinoma | Induces downregulation of FBXW7 | miR-367 targets FBXW7 mRNA 3'UTR directly and inhibits the expression of FBXW7 protein | (60) |
|  | non small cell lung cancer | Induces downregulation of FBXW7 | miR-367 targets FBXW7 mRNA 3'UTR directly and inhibits the expression of FBXW7 protein | (61) |
| miR-500a-3p | gastric cancer | Induces downregulation of FBXW7 | miR-500a-3p targets FBXW7 to decrease its expression, leading to the activation of CSCs properties and the resistance to docetaxel, cisplatin, and 5-FU | (63) |
| miR-586 | cervical cancer | Induces downregulation of FBXW7 | miR-586 targets FBXW7 mRNA 3'UTR directly and inhibits the expression of FBXW7 protein | (62) |
| miR-5000-3p | laryngeal cancer | Induces downregulation of FBXW7 | miR-5000-3p combines with FBXW7 and decreases the level of FBXW7 expression | (64) |

Supplementary Table 4: The long-non-coding RNA upregulates FBXW7. This table describes the lncRNA shows a positive effect on FBXW7. The lncRNA and their related cancer are indicated in the first and second columns. The results and pathway of their effects on FBXW7 are listed in the third and fourth columns.

| lncRNA | cancer | effect to FBXW7 | pathway | reference |
| --- | --- | --- | --- | --- |
| MIR22HG | laryngeal cancer | induces upregulation of FBXW7 | MIR22HG competitively binds to miR-5000-3p and induces higher level of FBXW7 expression by inhibiting the combination of miR-5000-3p and FBXW7 | (64) |
| MT1JP | gastric cancer | induces upregulation of FBXW7 | lncRNA MT1JP competitively binds to miR-92a-3p and induces higher level of FBXW7 expression by inhibiting the combination of miR-92a-3p and FBXW7 | (65) |
| MIF | cervical cancer | induces upregulation of FBXW7 | lncRNA MIF competitively binds to miR-586 and induces higher level of FBXW7 expression by inhibiting the combination of miR-586 and FBXW7 | (62) |
| CASC2 | hepatocellular carcinoma | induces upregulation of FBXW7 | lncRNA CASC2 competitively binds to miR-367 and induces higher level of FBXW7 expression by inhibiting the combination of miR-367 and FBXW7 | (60) |
| LINC00173 | cervical cancer | induces upregulation of FBXW7 | lncRNA LINC00173 competitively binds to miR-182-5p and induces higher level of FBXW7 expression by inhibiting the combination of miR-182-5p and FBXW7 | (56) |

Supplementary Table5：Other F-box proteins are also related to drug resistance in cancers.This table describes how the F-box proteins regulate drug sensitivity by degrading the corresponding substrates in various cancers. The drugs and therapy that can be affected are indicated in the first column. The cancer type and related F-box protein are listed in the second and third columns. The fourth column enumerates the way that how F-box protein regulates drug sensitivity.

| drug | cancer | F-box | pathway | reference |
| --- | --- | --- | --- | --- |
| cisplatin | non-small cell lung cancer | FBXO6 | FBXO6 degrades Chk1, reversing the cisplatin resistance | (85) |
| cisplatin | non-small cell lung cancer | FBXO22 | FBXO22 polyubiquites and degrades CD147, reversing the cisplatin resistance | (90) |
| cisplatin | cervical cancer | FBXL5 | FBXL5 and BTG3 inhibits the cell invasion and cisplatin chemoresistance | (91) |
| cisplatin | gastric cancer | FBXL5 | FBXL5 degrades ERK and p38, reversing the cisplatin resistance | (92) |
| camptothecin | non-small cell lung cancer | FBXO6 | Fbx6 degrades Chk1, reversing the camptothecin resistance | (84) |
| PARP inhibitors | breast cancer | FBXO5 | FBXO5 degrades RAD51, reversing the drug resistance | (83) |
| ibrutinib | mantle cell lymphoma | FBXWO10 | FBXWO10 degrades BCL-2, reversing the ibrutinib resistance | (86) |
| ionizing radiation (IR) | lung cancer | FBXL5 | FBXL5 degrades HSSB1 and activates ATM, reversing the drug resistance | (93) |
| paclitaxel | ovarian cancer | FBXL7 | FBXL7 degrades SURVIVIN, reversing the paclitaxel resistance | (95) |
